# P-1284. Phenotypic Mapping of Carbapenem-Resistant Gram-Negative Bacteria among Clinical Isolates in Nepal: High Prevalence of Combination NDM and OXA-48-like Carbapenemases, Absence of Plasmid-Mediated Colistin Resistance (mcr-1)

**DOI:** 10.1093/ofid/ofaf695.1472

**Published:** 2026-01-11

**Authors:** Prabhat Adhikari, Subha Shrestha, Balkrishna Awal, Sunita Gautam, Kabin Maleku, Ravindra m Sapkota, Suman Pant, Raju Bikram Basnet, Janak Koirala

**Affiliations:** Center for American Medical Specialists (CAMS), Nepal , Kathmandu, Bagmati, Nepal; Ceter for American Medical Specialists (CAMS), Kathmandu, Bagmati, Nepal; Ceter for American Medical Specialists (CAMS), Kathmandu, Bagmati, Nepal; Novala Biotech Pvt Ltd, Kathmandu, Bagmati, Nepal; Nepgen Clinical Research Services & Solutions, Kathmandu, Bagmati, Nepal; Novala Biotech Pvt Ltd, Kathmandu, Bagmati, Nepal; Nepgen Clinical Research Services & Solutions, Kathmandu, Bagmati, Nepal; KIST Medical College, Kathmandu, Bagmati, Nepal; Patan Academy of Health Sciences, Lalitpur, Bagmati, Nepal

## Abstract

**Background:**

Carbapenem-resistant Gram-negative bacteria (CR-GNB) threaten global health, especially in South Asia, where multidrug resistance (MDR) is driven by antibiotic overuse and poor infection control. Previous studies have reported a high prevalence of multidrug-resistant Gram-negative bacilli in Nepal, with widespread carbapenemase genes. The *mcr-1* gene, conferring colistin resistance, is reported in 1–21% of global samples and 20–60% of South Asian poultry, posing a pandrug-resistance risk. This study characterizes meropenem-resistant gram-negative bacteria in Nepal, assessing carbapenemase profiles, *mcr-1* status, and the possibility of other resistance mechanisms.

**Figure:**
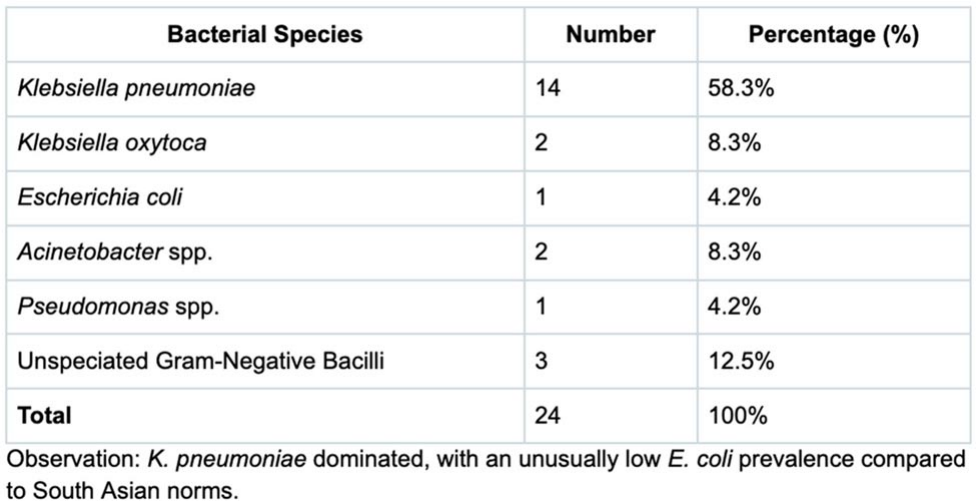
Distribution of Bacterial Species

**Figure:**
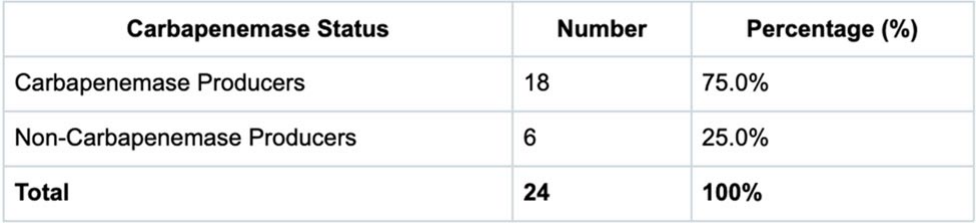
Carbapenemase Production

**Methods:**

A retrospective study at the Center for American Medical Specialists (CAMS) clinic, Nepal, analyzed 24 meropenem-resistant gram-negative bacteria from human clinical samples (blood, urine, sputum, wound swabs) collected May 2024–April 2025 from local tertiary hospitals. Carbapenemase production was tested using the FDA-approved NG Biotech CARBA 5 assay (KPC, OXA-48-like, NDM, VIM, IMP). 14 isolates were tested for *mcr-1* using NG Biotech’s MCR-1 assay.

**Figure:**
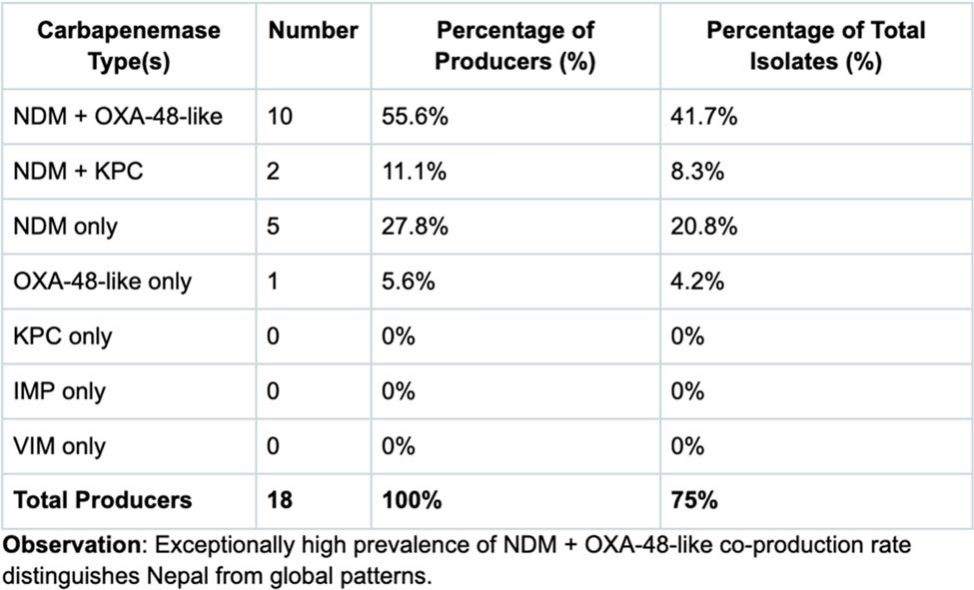
Distribution of Carbapenemase Types Among Producers

**Figure:**
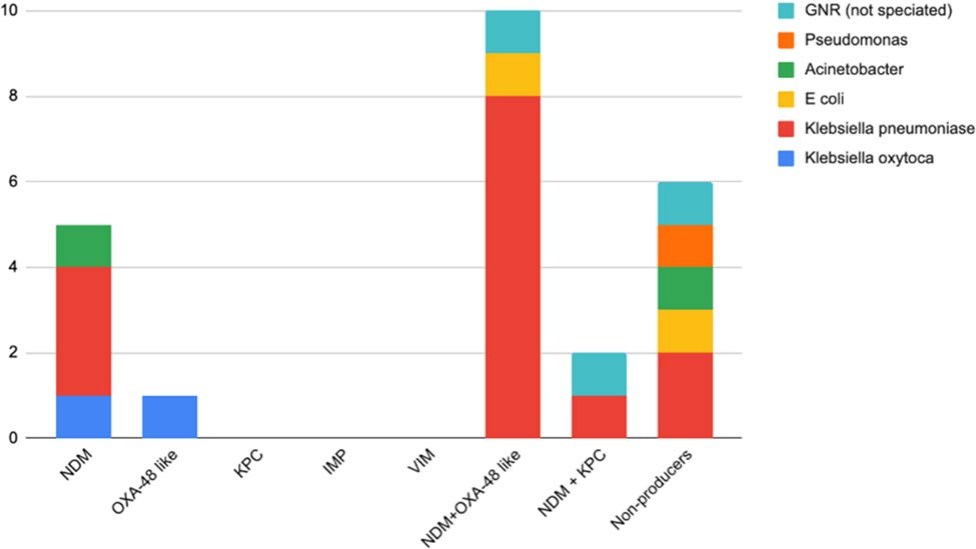
Phenotypic mapping of Carbapenem-Resistant Gram-Negative Bacteria

**Results:**

*K. pneumoniae* (58.3%) was the most prevalent CR-GNB. Carbapenemases occurred in 75% of isolates, with NDM being the dominant one at 94.4%; surprisingly, NDM + OXA-48-like (41.7%) was more prevalent than NDM alone (20.8%). One *Acinetobacter* isolate was meropenem-resistant, imipenem-sensitive, and carbapenemase-negative, suggesting efflux pumps or porin loss. All 14 tested isolates were *mcr-1*-negative. Details are shown in the tables.

**Conclusion:**

This study's 75% carbapenemase rate and 41.7% NDM + OXA-48-like co-production reflect South Asia’s MDR crisis, surpassing USA/Europe rates. Although a small sample size, the low *E. coli* prevalence (4.2%) in this study is atypical for South Asia. The absence of *mcr-1* in clinical isolates, despite its prevalence in regional poultry, may preserve colistin’s efficacy. Point-of-care diagnostics are vital for Nepal’s resource-limited setting, aiding targeted therapy, infection control, and antimicrobial stewardship.

**Disclosures:**

Prabhat Adhikari, MD (Infectious Diseases & Critical Care), Novala Biotech Pvt Ltd, Nepal: Advisor/Consultant|Novala Biotech Pvt Ltd, Nepal: Board Member Sunita Gautam, PhD (Molecular Biotechnology), Novala Biotech Pvt Ltd, Nepal: Advisor/Consultant|Novala Biotech Pvt Ltd, Nepal: Board Member Kabin Maleku, Pharmacist (MPharm), Novala Biotech Pvt Ltd, Nepal: Advisor/Consultant|Novala Biotech Pvt Ltd, Nepal: Board Member Ravindra m. Sapkota, PhD (Immunology), Novala Biotech Pvt Ltd, Nepal: Advisor/Consultant|Novala Biotech Pvt Ltd, Nepal: Board Member

